# A C9ORF72 BAC mouse model recapitulates key epigenetic perturbations of ALS/FTD

**DOI:** 10.1186/s13024-017-0185-9

**Published:** 2017-06-12

**Authors:** Rustam Esanov, Gabriela Toro Cabrera, Nadja S. Andrade, Tania F. Gendron, Robert H. Brown, Michael Benatar, Claes Wahlestedt, Christian Mueller, Zane Zeier

**Affiliations:** 1 Department of Psychiatry & Behavioral Sciences, Center for Therapeutic Innovation, University of Miami Miller School of Medicine, 1501 NW 10th Ave, BRB-413, Miami, FL 33136 USA; 2 Department of Neurology, University of Massachusetts Medical School, Worchester, MA 016555 USA; 3 Department of Neuroscience, Mayo Clinic, Jacksonville, FL 32224 USA; 4 Department of Neurology, University of Miami Miller School of Medicine, Miami, FL 33136 USA; 5 Department of Pediatrics and Horae Gene Therapy Center, University of Massachusetts Medical School, Worchester, MA 016555 USA; 6 Department of Pediatrics and Horae Gene Therapy Center, University of Massachusetts Medical School, 368 Plantation Street, AS6-2053, Worcester, MA 01605 USA

**Keywords:** Amyotrophic lateral sclerosis, C9ORF72 BAC mouse, Induced pluripotent stem cells, DNA methylation, Hydroxymethylation, R-loops

## Abstract

**Background:**

Amyotrophic Lateral Sclerosis (ALS) is a fatal and progressive neurodegenerative disorder with identified genetic causes representing a significant minority of all cases. A GGGGCC hexanucleotide repeat expansion (HRE) mutation within the C9ORF72 gene has recently been identified as the most frequent known cause of ALS. The expansion leads to partial heterochromatinization of the locus, yet mutant RNAs and dipeptide repeat proteins (DPRs) are still produced in sufficient quantities to confer neurotoxicity. The levels of these toxic HRE products positively correlate with cellular toxicity and phenotypic severity across multiple disease models. Moreover, the degree of epigenetic repression inversely correlates with some facets of clinical presentation in C9-ALS patients. Recently, bacterial artificial chromosomes (BAC) have been used to generate transgenic mice that harbor the HRE mutation, complementing other relevant model systems such as patient-derived induced pluripotent stem cells (iPSCs). While epigenetic features of the HRE have been investigated in various model systems and post-mortem tissues, epigenetic dysregulation at the expanded locus in C9-BAC mice remains unexplored.

**Methods and Results:**

Here, we sought to determine whether clinically relevant epigenetic perturbations caused by the HRE are mirrored in a C9-BAC mouse model. We used complementary DNA methylation assessment and immunoprecipitation methods to demonstrate that epigenetic aberrations caused by the HRE, such as DNA and histone methylation, are recapitulated in the C9-BAC mice. Strikingly, we found that cytosine hypermethylation within the promoter region of the human transgene occurred in a subset of C9-BAC mice similar to what is observed in patient populations. Moreover, we show that partial heterochromatinization of the C9 HRE occurs during the first weeks of the mouse lifespan, indicating age-dependent epigenetic repression. Using iPSC neurons, we found that preventing R-loop formation did not impede heterochromatinization of the HRE.

**Conclusions:**

Taken together, these observations provide further insight into mechanism and developmental time-course of epigenetic perturbations conferred by the C9ORF72 HRE. Finally, we suggest that epigenetic repression of the C9ORF72 HRE and nearby gene promoter could impede or delay motor neuron degeneration in C9-BAC mouse models of ALS/FTD.

**Electronic supplementary material:**

The online version of this article (doi:10.1186/s13024-017-0185-9) contains supplementary material, which is available to authorized users.

## Background

A pathological GGGGCC hexanucleotide repeat expansion mutation located within the non-coding portion of the C9ORF72 gene has been identified as the cause of chromosome 9p21-linked ALS and frontotemporal dementia (FTD) [1, 2]. The discovery places ALS and FTD among a large family of nearly 40 repeat expansion disorders, many of which specifically affect neurons [3]. The C9ORF72 HRE is hypothesized to confer cytotoxicity, in part, via RNA gain of function whereby sense and antisense transcripts harboring the repeat sequester RNA binding proteins resulting in ribonucleoprotein foci [4, 5], preventing the proteins from carrying out their normal function. In addition, expanded RNAs and dipeptide-repeat proteins that arise from aberrant translation of mutant RNAs disrupt nucleocytoplasmic transport [6–8]. Given that mutant RNAs and the DPRs derived from them are the primary source of pathology in C9-ALS and epigenetic mechanisms regulate their production, it follows that epigenetic regulation of expanded C9ORF72 alleles is of particular significance.

Epigenetic alterations occur in many repeat expansion disorders and there is now definitive evidence that epigenetic perturbations associated with the C9ORF72 HRE contribute to C9-ALS pathophysiology [9–11]. Expanded C9ORF72 alleles have reduced transcription rates, are depleted of active histone marks with concomitant enrichment of repressive epigenetic marks, including DNA hypermethylation in some cases. Specifically, the levels of all three C9ORF72 transcript variants are reduced in C9-ALS, including variant 2 which does not contain the repeat sequence due to alternative transcription start site (TSS) utilization [1, 5, 12]. Enriched repressive epigenetic marks include tri-methylation of histone 3 at lysine positions 9 and 27 (H3K9me3, H3K27me3) and histone 4 lysine 20 (H4K20me3) [12]. In addition, DNA methylation of cytosine residues within CpG islands near the C9ORF72 promoter occurs in approximately 30% of patients [13–15]. Notably, promoter hypermethylation is theorized to be protective due to decreased production of toxic products in patient cells [16] leading to reduced neuronal loss in the brain [17]. Mechanistically, DNA hypermethylation is counteracted by active DNA demethylation [18] while repeat instability is conferred by highly stable GC-rich RNA-DNA duplex formation across the HRE [19]. In addition to repeat instability, RNA-DNA hybrid structures, or R-loops, may also contribute to C9ORF72 hypermethylation. Indeed, R-loops are known to regulate DNA methylation at CpG islands of gene promoters including those affected by repeat expansion mutations [20, 21]. Taken together, these observations indicate that the C9ORF72 HRE alters the local epigenetic environment such that the rate of transcription, DNA methylation, histone modification and R-loop formation are all perturbed by the expansion mutation.

Two independent groups were first to develop transgenic mouse models of C9-ALS carrying the pathogenic C9ORF72 HRE [22, 23]. The human repeat expansion sequence was introduced into the mouse genome using a bacterial artificial chromosome (BAC). The C9-BAC mice displayed typical histopathological gain-off-function features of C9-ALS including RNA foci and DPR aggregates, yet no motor or cognitive phenotypes were observed. Subsequently, two additional groups generated C9-BAC mice that exhibit reduced survival, motor deficits and cognitive dysfunction [24, 25]. While these previous reports focused on the production of toxic HRE products, none have described epigenetic features of the C9ORF72 transgene. Notably, the number of toxic mRNA foci and DPR abundance in C9-BAC mice was observed to significantly decline as a function of age [23]. This is somewhat counter-intuitive since ALS/FTD are age-related disorders. Therefore, we hypothesized that partial epigenetic repression of the C9ORF72 transgene could potentially attenuate the severity of phenotypes in C9-BAC mice, limiting their applicability to human disease. Herein, we performed a post-natal developmental study using transgenic BAC mice reported by Peters et al. [23] to systematically investigate whether characteristic epigenetic features of the HRE in C9-ALS are recapitulated in this mouse model system.

## Methods

### C9-BAC mice

Animals used in the present study have previously been described [23]. Briefly, a BAC containing human sequence from a C9-ALS patient, spanning the gene promoter to exon 6, was inserted into the mouse genome using standard transgenic methods. All experimental procedures involving transgenic mice were performed in accordance with the guidelines of Institutional Animal Care and Use Committee of the University of Massachusetts Medical School.

### DNA and RNA extraction

Genomic DNA and total RNA extractions were performed from 30 mg of mouse brain tissues using a kit (AllPrep DNA/RNA Mini Kit®, Qiagen #80204) as per manufacturers’ instructions.

### Quantitative PCR (qPCR)

Reactions were performed using TaqMan® assays that amplify C9ORF72 transcript variants V1, V2, V3 (Hs00376619_m1), C9ORF72 V1, V3 (Hs00331877_m1), C9ORF72 V2 (NM_018325.3, L/R/Probe: 5’CGGTGGCGAGTGGATATCTC / 5’TGGGCAAAGAGTCGACATCA/ 5’TAATGTGACAGTTGGAATGC), mouse Glyceraldehyde 3-phosphate dehydrogenase (GAPDH) (Mm99999915_g1), beta-actin (Mm00607939_s1), eukaryotic 18S ribosomal RNA (Hs99999901_s1) and human GAPDH (Hs02758991_g1). Relative quantification values were calculated using a standard curve method, normalized to the average of GAPDH and 18S endogenous control genes.

### Digital droplet PCR (ddPCR)

For gene expression, 500 ng of total RNA was used in a 20 μl reaction to generate complementary DNA (cDNA) using random hexamer primers and MultiScribe reverse transcriptase (High capacity RNA-to-cDNA Kit, Life Technologies). After diluting the cDNA 1:1.25, 2 μl were loaded into a 20 μl reaction containing ddPCR Supermix for Probes (nodUTP) (Bio-Rad, Gladesville, NSW, Australia), and 1 μl of primers amplifying all three C9ORF72 transcripts (Hs00376619_m1, Life Technologies). The 20 μl sample, along with 70 μl of ddPCR-oil was loaded into a DG8 cartridge and covered with a gasket according to manufacturer’s instructions; the cartridge was then placed in a QX100 droplet generator. 40 μl of sample were then transferred to a 96-well PCR plate and put in a normal PCR thermocycler (Eppendorf, North Ryde, NSW, Australia). The plate was then placed in the QX100 ddPCR reader (Bio-Rad, Gladesville, NSW, Australia) for absolute quantification.

### Methylation-sensitive quantitative PCR

DNA methylation at the C9ORF72 promoter was assessed using previously reported methods [26]. Briefly, 100 ng of genomic DNA was used for methylation-sensitive restriction digest with four units of HhaI endonuclease. Two sets of primers were used for subsequent quantitative PCR: C9me (L/R: 5’CGGTAAAAACAAAATTTCATCCA / 5’GGGCAACTTGTCCTGTTCTT) spans two HhaI restriction sites within the C9ORF72 promoter, and C9ec (L/R: 5’AGGAAAGAGAGGTGCGTCAA / 5’TCCTAAACCCACACCTGCTC) was used as an endogenous control.

### Bisulfite pyrosequencing

Genomic DNA (2 μg) was treated with bisulfite and analyzed by pyrosequencing (EpigenDx Inc.) as previously reported [18]. The DNA methylation assessment was performed using the PSQTM96HS system and custom assays developed by EpigenDx (ADS3232-FS1 and ADS3232-FS2). The assay covers 16 cytosine residues within the C9ORF72 promoter from −55 to +125 base pairs relative to the C9ORF72 transcriptional start site.

### Copy Number Variation (CNV) analysis

C9ORF72 mouse brains were fresh frozen and cut in a sagittal section to extract DNA. The tissue was lysed overnight at 55 °C, RNAse treated and eluted following manufacturer’s instructions (Gentra Puregene Tissue Kit, Qiagen). The DNA was diluted to 20 ng/μl and 2 μl were added to the ddPCR master mix along with 1 μl of each primer/probe. Primer and probe sequences used for human genomic C9ORF72 (L/R/Probe: 5’AAGGCACAGAGAGAATGGAAG / 5’AGGCTTATTCGTATGTCTCCAAG / 5’AGGTTGATGGCTACATTTGTCAAGGC) and for mouse EIF2C1 diploid genome (L/R/Probe: 5’CCTGCCATGTGGAAGATGAT / 5’GAGTGTGGTGGCTGGATTTA / 5’TGGGGAGAGCTGGAGCCAG) quantification are indicated. Droplets were generated followed by PCR and absolute quantification.

### Poly(GP) immunoassay

C9-BAC whole brain tissues were lyzed in buffer containing 50 mM Tris–HCl, pH 7.4, 300 mM NaCl, 1% Triton X-100, 2% sodium dodecyl sulfate, 5 mM EDTA, as well as protease (EMD Millipore) and phosphatase inhibitors (Sigma-Aldrich). After sonication, samples were centrifuged at 16,000 x g for 20 min at 4 °C and supernatants collected. The protein concentration of lysates was determined by BCA assay (Thermo Scientific). Poly(GP) levels in lysates were measured using a previously described sandwich immunoassay that utilizes Meso Scale Discovery (MSD) electrochemiluminescence detection technology [27]. Lysates were diluted in Tris-buffered saline (TBS) and tested using 43 μg of protein per well in duplicate wells. Serial dilutions of recombinant (GP)_8_ in TBS were used to prepare the standard curve. Response values corresponding to the intensity of emitted light upon electrochemical stimulation of the assay plate using the MSD QUICKPLEX SQ120 were acquired for interpolation of poly(GP) levels using the standard curve.

### Generation of C9ORF72 depleted induced pluripotent stem cell lines

Previously, we generated several patient-derived iPSC lines from C9ORF72 ALS patients [18]. One of these lines was transduced with a lentiviral vector (Sigma-Aldrich, SHCLNV-NM-018325, clone TRCN0000148881) expressing a puromycin resistance gene and a small hairpin RNA (shRNA) targeting all three C9ORF72 transcript variants. Transduction of iPSCs (MOI = 3) was carried out in the absence of polybrene. Selection was performed in the presence of puromycin (1 μg/ml) for 10 days; surviving colonies were expanded and used for motor neuronal differentiation. Depletion of C9ORF72 RNAs was confirmed by qPCR.

### Motor neuron differentiation

Motor neuronal differentiation was performed according to our previously published protocol [26]. Briefly, neural precursor spheres were differentiated from iPSC colonies using neural induction medium and cultured in ultra-low attachment flasks. Afterwards, spheres were seeded onto poly-L-ornithine and laminin-coated plates with neuronal induction medium and cultured for 4 weeks to obtain terminally differentiated motor neurons.

### DNA-RNA immunoprecipitation (DRIP)

The DRIP protocol was adapted from Loomis et al. [28] with some modifications. Here, 5 × 10^6^ cells were lysed with 0.5% SDS and treated with 400 units of proteinase K at 37 °C overnight. The next day, DNA extraction was performed using standard phenol/chloroform/isopropanol precipitation. Fifty micrograms of DNA were digested with EcoR1, HindIII, BsrGI, and XbaI (20 units each) at 37 °C for 1 h. Next, samples were incubated in the presence of the S9.6 antibody (Kerafast ENH001) for 2 h at 4 °C with inversion. As a control, samples were treated with 25 units of RNase H (ThermoFisher Scientific #EN0201) for 6 h at 37 °C. Following DRIP, DNA was precipitated with isopropanol and used for quantitative PCR with primers amplifying a region upstream (L/R: 5’AAGAGCAGGTGTGGGTTTAG / 5’GAGTACTGTGAGAGCAAGTAGTG) and downstream (L/R: 5’CTCAGAGCTCGACGCATTT / 5’CAATTCCACCAGTCGCTAGA) of the C9ORF72 hexanucleotide repeat expansion.

### C9ORF72 GGGGCC repeat methylation assessment

To measure C9ORF72 HRE methylation, we combined HpaII methylation-sensitive restriction digest with the repeat-primed PCR assay in the presence of 5% dimethyl sulfoxide and complete substitution of 7-deaza-2-deoxy GTP for dGTP as previously described [18]. PCR fragments were analyzed using the ABI3730 DNA Analyzer and GeneMapper software. The area under the curve (AUC) values were calculated for peaks higher than 150 base pairs. The AUC of digested samples were normalized to AUC of uncut controls to calculate the percent DNA methylation of the HRE sequence.

### DNA hydroxymethylation assessment

To quantify 5hmC and 5mC levels at the C9ORF72 promoter, we utilized the EpiMark® 5-hmC and 5-mC Analysis Kit (New England Biolabs E3317), according to the manufacturer’s protocol as previously reported [18]. Briefly, 2 μg of genomic DNA was subjected to glycosylation by T4 β-glucosyltransferase, converting 5hmC to 5ghmC residues. Glucosylated DNA was then digested with MspI or HpaII endonucleases for 6 h at 37 °C and subsequently amplified by quantitative PCR using the -313 bp primer set (L/R: 5’AGGAAAGAGAGGTGCGTCAA / 5’TCCTAAACCCACACCTGCTC) and +104 bp primer set (L/R: 5’AAATTGCGATGACTTTGCAG / 5’ACTGCAAACCCTGGTAGG), each of which spans one CCGG MspI/HpaII restriction site.

### Chromatin immunoprecipitation (ChIP)

Immunoprecipitation of H3K9me3 was performed as we previously reported [26]. Briefly, 50 mg of brain tissue was sliced into small pieces and crosslinked in 1% formaldehyde for 10 min and quenched with 0.125 M glycine for 5 min at room temperature. Sonication was performed for 5 min using the Bioruptor UCD200 sonication system set to the high setting. Lysates were then transferred to a tube containing Protein G Dynabeads prepared with an anti-H3K9me3 antibody (Abcam ab8898), or control rabbit IgG, and incubated overnight at 4 °C with rotation. The following day, stringency washes were performed and samples were re-suspended in 150 μL elution buffer consisting of 1% SDS, 0.1 M NaHCO_3_, 0.2 M NaCl and incubated at 65 °C overnight. The next day, samples were de-crosslinked by treatment with 50 μL proteinase K and incubated at 42 °C for 2 h. De-crosslinked genomic DNA was isolated using the QiaQuick PCR purification kit (Qiagen #28104) according to the manufacturer’s instructions. Quantitative PCR was performed using two primer sets: C9.A (L/R: 5’ACTCGCTGAGGGTGAACAAG / 5’TCCTGAGTTCCAGAGCTTGC) and C9.B (5’AAGAGCAGGTGTGGGTTTAG / 5’GAGTACTGTGAGAGCAAGTAGTG).

## Results

### Expression of the human C9ORF72 transgene decreases while H3K9 tri-methylation increases in C9-BAC mice during the first post-natal weeks

**Fig. 1:**
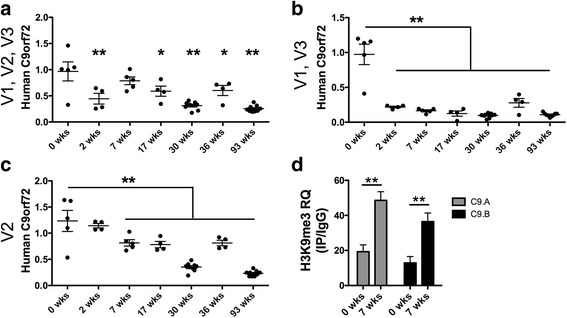
C9ORF72 transcription decreases while a repressive histone methylation mark increases in the brain of C9-BAC mice during the first post-natal weeks. Values of human C9ORF72 in the BAC mouse cortex, normalized to the average of GAPDH and 18S, are shown for primers amplifying transcript variants V1, V2, V3 (**a**); V1, V3 (**b**) and V2 (**c**). Age groups are indicated on the x-axis in weeks (wks). Mean and standard error of the mean (SEM) are indicated by long and short bars respectively. For each primer set, a one-way analysis of variance was performed (*p* < 0.001). Bonferroni’s multiple comparison test was performed between neonatal (0wks) and the rest of the age groups, significance is indicated by *p* < 0.05 * and *p* < 0.01 **. H3K9me3 levels were assessed by chromatin immunoprecipitation in brain tissues from 0 to 7 week-old C9-BAC mice (*n* = 5). Two different primer sets were used: C9.A and C9.B amplifying regions within the human C9ORF72 promoter. Relative enrichment of H3K9me3 was calculated by real-time PCR amplification of immunoprecipitants (IP) relative to inputs and an IgG negative control IP, *p* < 0.01 ** (**d**)

Tissue samples from mice of seven different age groups were assessed in this study: post-natal week 0 (*n* = 5), 2 (*n* = 4), 7 (*n* = 5), 17 (*n* = 4), 30 (*n* = 9), 36 (*n* = 4) and 93 (*n* = 9). The activity and epigenetic status of the human C9ORF72 transgene was assessed by quantifying levels of mRNAs and histone methylation within the promoter of the human gene. First, we measured human C9ORF72 mRNA levels in the neocortex of C9-BAC mice across all age groups using quantitative real-time (qPCR). Three primer sets were used to amplify different human C9ORF72 mRNA transcript variants (V1, V2, and V3) as well as three endogenous controls (beta-actin, GAPDH and 18S). Due to the observed variability in beta-actin expression levels across age groups (Additional file 1: Figure S1A), we used the average values of GAPDH and 18S for normalization. We found that levels of all three C9ORF72 transcript variants are significantly reduced, starting within the first post-natal weeks of age (Fig. 1). To confirm our findings, we performed digital droplet quantitative PCR (ddPCR) for absolute quantification of the human C9ORF72 levels and observed a similar trend (Additional file 1: Figure S1B). This observation suggests that partial epigenetic repression of the C9ORF72 transgene in the brain of post-natal C9-BAC mice is age-dependent. Next, we utilized chromatin immunoprecipitation (ChIP) to isolate H3K9me3-bound DNA fragments from brain samples of 0 and 7 weeks-old C9-BAC mice. H3K9me3 is a repressive epigenetic mark that negatively correlates with transcription rates and is enriched within the promoter region of expanded C9ORF72 alleles as compared to unexpended alleles [12, 26, 29]. Using two different primer sets (C9.A and C9.B), we found that H3K9me3 levels within the promoter of the human C9ORF72 transgene were significantly increased in the brain of C9-BAC mice by week 7 (Fig. 1d). Increased H3K9me3 indicates partial epigenetic repression of the mutant gene locus occurs in the first post-natal weeks of life.

### DNA hypermethylation within the promoter of the human C9ORF72 transgene occurs in a subset of C9-BAC mice, similar to the human C9-ALS patient population

**Fig. 2:**
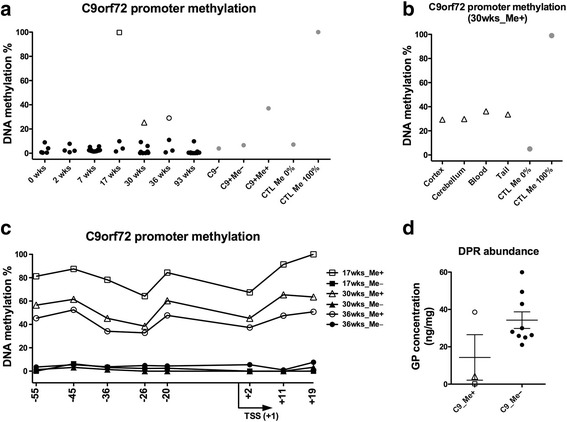
DNA hypermethylation at the expanded C9ORF72 promoter appears in a fraction of adult mice. Site-specific DNA methylation sensitive PCR assessment of the human C9ORF72 promoter in the cortex of C9-BAC mice at seven time points, indicated in weeks (wks) of age. Two HhaI restriction sites located at −215 and −109 base pairs from the transcriptional start site were interrogated; three hypermethylated animals are indicated by *open shapes* (17wks square, 30wks triangle and 36wks circle). Assay controls (*grey circles on right*) include DNA isolated from post mortem brain tissues of ALS patients with the hexanucleotide repeat expansion (C9+) with (me+) or without (me-) promoter hypermethylation, an unaffected healthy control (C9-) individual, and synthetic DNA enriched (CTL Me 100%) or depleted of 5mC (CTL Me 0%). Values are plotted relative to the synthetic high control, which is set to 100% (**a**). C9ORF72 promoter methylation assessment from brain cortex, cerebellum, blood and tail clippings of a 30 week old hypermethylated mouse using HhaI methylation sensitive PCR (**b**). Bisulfite pyrosequencing of brain cortex from 17, 30 and 36 weeks old C9-BAC mice (*n* = 1 per age group per methylation status) across 8 CpG dinucleotides within the human C9ORF72 promoter, positions relative to TSS are shown on the x-axis. *Open symbols* indicate samples from hypermethylated (me+) animals, filled symbols are samples from unmethylated (me-) animals (**c**). Glycine-Proline DPR assessment of whole brain tissue samples from three hypermethylated animals (*open symbols*) and representative unmethylated samples (*filled symbols*) from 17, 30 and 36 week old C9-BAC mice (*n* = 3 per age group) (**d**)

In about 30% of all C9-ALS cases, methylated CpG dinucleotides within the C9ORF72 promoter occur more frequently [13, 15]. This promoter hypermethylation is associated with a modestly attenuated phenotype indicating that epigenetic repression is clinically relevant [16, 17]. To assess levels of DNA methylation within the promoter of the C9ORF72 transgene, we extracted DNA from the cortex of C9-BAC mice and performed methylation-sensitive PCR as previously reported [26]. Interestingly, we found that in three adult mice, the C9ORF72 promoter was hypermethylated, similar to what occurs in a subset of human C9-ALS patients (Fig. 2a). Similar to C9-ALS patients, C9ORF72 promoter hypermethylation in C9-BAC mice was stable across different brain regions (cortex and cerebellum) and somatic tissues (blood and tail clippings) (Fig. 2b). To confirm these findings, we utilized a secondary method to assess DNA methylation. Using bisulfite pyrosequencing, we measured DNA methylation across eight different CpG dinucleotides located both upstream and downstream of the transcription start site (TSS) of the human C9ORF72 promoter (Fig. 2c). Furthermore, we have eliminated the possibility that increased methylation levels are due to the abnormal copy number of the human C9ORF72 transgene by CNV analysis (Additional file 2: Figure S2). Our data demonstrate that adult mice from the same age group can have differential DNA methylation levels within the C9ORF72 promoter, mirroring a phenomenon observed in C9-ALS patients. To determine whether promoter hypermethylation affects the production of toxic DPRs, we next measured levels of glycine-proline (GP) repeat proteins in the brains of three hypermethylated and nine unmethylated C9-BAC mice (Fig. 2d). Our data indicate that there is a trend towards decreased GP levels in hypermethylated mice. Nevertheless, further studies with more animals are required to fully investigate the effect of DNA hypermethylation at the C9ORF72 promoter on DPR production in these mice. Notably, GP levels in hypermethylated mice negatively correlated with the DNA methylation percentile as determined by bisulfite pyrosequencing analysis of 8 CpG dinucleotides (Additional file 3: Figure S3). The goodness of fit (R^2^) was similar for each of the 8 CpGs suggesting none have a more prominent regulatory role than others.

### Methylation of GGGGCC repeat expansion mutation increases with age in C9-BAC mice

**Fig. 3:**
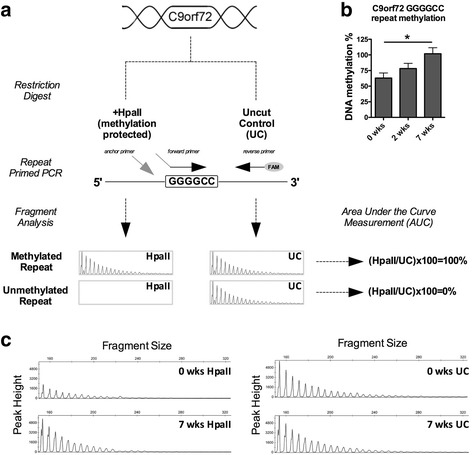
Hexanucleotide (GGGGCC) repeat methylation increases with age in C9-BAC mice. An illustration of the HRE methylation assessment method. For each sample, two repeat-primed PCR reactions are performed, one using DNA subjected to HpaII digest and one using uncut control (UC). DNA methylation percentage is calculated based on the area under the curve values following capillary fragment analysis of HpaII digested samples normalized to the uncut control sample (**a**). HRE methylation levels were assessed at 0 (*n* = 5), 2 (*n* = 4) and 7 (*n* = 5) weeks of age, significance is indicated by *p < 0.05 (**b**). Representative electropherograms from 0 to 7 week old C9-BAC mouse brain cortex DNA treated with HpaII as well as uncut control (**c**)

Previously, Xi et al. reported that the HRE itself is methylated in every C9-ALS patient with the pathogenic repeat number [30], indicating a protective cellular response. Interestingly, the authors argue that the origin of DNA hypermethylation initiates within the HRE, but in some individuals, methylation spreads upstream to the promoter region. Therefore, we sought to determine if HRE hypermethylation is recapitulated in a C9-BAC mouse model of ALS. To quantitatively assess DNA methylation levels at the HRE in C9-BAC mice, we developed a novel assay that combines the HpaII methylation-sensitive restriction digest, which recognizes CCGG sequence, with repeat-primed PCR and capillary electrophoresis (Fig. 3a). Given our finding that expression of the human C9ORF72 transgene decreases within the first weeks of life (Fig. 1), we quantified DNA methylation of the HRE at 0, 2 and 7 weeks of age (Fig. 3b). We observed that HRE methylation was present in all three age groups but increased with age, reaching significance between 0 and 7 weeks. Representative graphs from one animal per group are shown in Fig. 3c. These findings suggest that methylation of cytosine residues within the HRE sequence itself occurs within the first weeks of the C9-BAC mouse lifespan.

### Hydroxymethylcytosine is observed at the expanded C9ORF72 promoter in the brain of C9-BAC mice

**Fig. 4:**
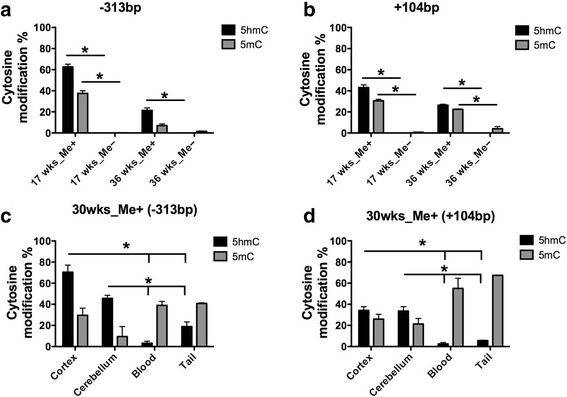
DNA demethylation is observed at the expanded C9ORF72 promoter distinctively in the brain. Two CpG dinucleotides located within MspI/HpaII restriction sites at positions −313 and +104 base pairs from the C9ORF72 transcriptional start site were interrogated by 5-methylcytosine (5mC) and 5-hydroxymethylcytosine (5hmC) sensitive PCR. The y-axis indicates percent 5hmC (*black*) and 5mC (*grey*) from brain cortex samples for a subset of C9-BAC mice (**a**, **b**), error bars represent standard deviation, experiments were performed in duplicates (*N* = 2 from a single biological sample for each age and methylation status). Assessment of 5hmC enrichment at two restriction sites across tissue types of a 30 week old hypermethylated mouse are illustrated in **c** and **d**. Student’s t-test was performed to determine significance, indicated by *p* < 0.05 *

We previously reported that 5-hydroxymethylcytosine (5hmC), a stable intermediate of the DNA demethylation pathway, is a novel epigenetic feature of repeat expansions mutations in Fragile X Syndrome and C9-ALS [18, 31]. Here, we sought to determine whether 5hmC is present at the C9ORF72 promoter of hypermethylated BAC mice as well. We performed DNA hydroxymethylation assessment as previously described [18, 31]. We found that similar to C9-ALS patients, hypermethylated C9-BAC mice show elevated levels of 5hmC at the C9ORF72 promoter sites both upstream and downstream of the TSS in the cortex (Fig. 4a, b). Furthermore, we assessed peripheral tissues from the 30 week-old hypermethylated mouse. We found that 5hmC enrichment was unique to brain tissues (Fig. 4c, d). This indicates more pronounced DNA demethylation in the brain of hypermethylated C9-BAC mice which is consistent with the highest abundance of the global 5hmC levels occurring in the central nervous system as compared to other tissue types, presumably due to the higher ten-eleven-translocation (TET) enzyme activity [32, 33].

### DNA hypermethylation is acquired independently of RNA-DNA hybrid formation at the C9ORF72 locus

**Fig. 5.**
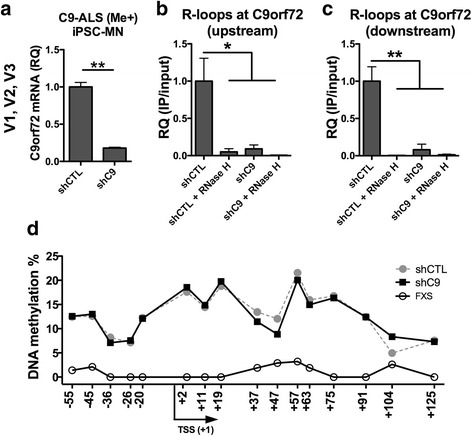
DNA methylation is acquired independently of RNA-DNA hybrid formation at the C9ORF72 locus. Relative quantification of all three C9ORF72 transcript variants in iPSC-derived motor neurons stably expressing a C9ORF72-specifc shRNA (shC9) or a scrambled CTL (shCTL) (**a**). DNA-RNA immunoprecipitation at the C9ORF72 promoter of shC9 and shCTL motor neurons, relative quantification was measured using two sets of primers, designed upstream (**b**) and downstream (**c**) of the repeat expansion, RNase H treatment was performed prior to pull-down as a negative control. DNA methylation levels at the C9ORF72 promoter were assessed using bisulfite pyrosequencing across 16 CpG dinucleotides; positions relative to the transcription start site are indicated on the x-axis (**d**). Fragile X patient-derived iPSC-neurons (FXS) were used as a negative control. All experiments were performed in duplicates (*N* = 2 from a single biological sample for each iPSC line examined). Significance is indicated by *p* < 0.05 * and *p* < 0.01 **

We next sought to investigate one possible mechanism of DNA methylation acquisition at the C9ORF72 promoter of ALS patients. By analogy to other repeat expansion disorders, such as Fragile X Syndrome and Freidriech’s Ataxia [21, 34], we hypothesized that DNA-RNA hybrids or R-loops formed by an expanded C9ORF72 transcript lead to epigenetic silencing. In our previous report, we demonstrated that iPSC lines from a hypermethylated ALS patient can be used as a tool to investigate the acquisition of DNA methylation at the C9ORF72 promoter [18]. In particular, we showed that DNA methylation at the C9ORF72 promoter is erased during iPSC generation and then re-acquired during neuronal differentiation. To determine whether C9ORF72 promoter DNA hypermethylation occurs via DNA-RNA hybrid formation, we created a stable iPSC line from a hypermethylated ALS patient expressing a small hairpin RNA targeting all three C9ORF72 transcript variants (shC9) or a scrambled control (shCTL). We reasoned that disrupting R-loop formation by depleting C9ORF72 mRNAs in iPSCs would prevent DNA hypermethylation upon neuronal differentiation, similar to what was previously shown in Fragile X Syndrome [34]. We generated stable iPSC cell lines expressing either the shC9 or shCTL constructs then differentiated the cells into motor neurons using our previously published protocols [18]. We confirmed efficient depletion of C9ORF72 in shC9 iPSC lines using qPCR (Fig. 5a). We then confirmed efficient disruption of R-loop formation at the C9ORF72 locus in shC9 motor neurons using DNA-RNA immunoprecipitation (DRIP) followed by qPCR with primers to amplify regions upstream and downstream the HRE (Fig. 5b, c). Finally, DNA methylation at the C9ORF72 promoter in motor neurons was assessed across 16 individual CpG dinucleotides using bisulfite pyrosequencing (Fig. 5d). Patient-derived motor neurons from an individual with the Fragile X Syndrome that does not harbor C9ORF72 repeat expansion were used as a negative control. Despite efficient knockdown of C9ORF72 RNAs and disruption of R-loops, we did not observe significant differences in DNA methylation levels at the C9ORF72 promoter between shC9 and shCTL motor neurons. These results suggest that R-loops may not be required for the acquisition of DNA hypermethylation associated with the C9ORF72 HRE.

## Discussion

Here we report that C9-BAC mice from Peters et al. [23] recapitulate epigenetic perturbations seen in C9ORF72-associated ALS and FTD patients. We observed enrichment of the repressive epigenetic mark H3K9me3 in the promoter of the human transgene, which corresponded to decreased transcription rates. We also observed DNA methylation within the HRE repeat sequence itself for all mice tested and in three mice, this methylation had spread to the promoter region. Our data show that HRE-mediated epigenetic repression occurs during the first few weeks after birth in C9-BAC mice and likely occurs at about the time of birth in humans (2 weeks in the mouse), although the unavailability of human tissues makes this difficult to assess. It will be important to determine whether epigenetic features of the HRE are shared among different C9-BAC lines. Of particular interest will be to determine whether phenotypic severity is associated with levels of epigenetic repression, in cases where a phenotype is observed, and whether the epigenetic repression is lost (even relatively) as animals continue to age and develop a clinical phenotype.

Our findings demonstrate that epigenetic features of the HRE in humans are mirrored in the C9-BAC mouse, and in some cases, appear at a similar levels and frequencies. For example, DNA hypermethylation of the HRE itself in C9 ALS/FTD patients occurs at a rate of 97% [30]. Using a quantitative assay to assess HRE methylation, we found that all C9-BAC mice assessed exhibit hypermethylation of the repeat sequence. This HRE methylation increases with age, although P0 mice also show elevated levels of methylation within the repeat. These observations support the hypothesis that increased levels of DNA methylation within the repeat precede promoter hypermethylation and is a common feature of the HRE that serves a protective role to impede transcription across the mutation [16, 17, 30, 35]. The C9ORF72 promoter hypermethylation on the other hand, is less common in C9-ALS patients, and is only observed in a fraction (~30%) of individuals with a pathogenic repeat number [13, 15]. Interestingly, we found that to be true for C9-BAC mice as well, where only a small fraction of the adults show DNA hypermethylation at the C9ORF72 promoter, across different tissues. This is particularly poignant given the neuroprotective effect of the C9ORF72 promoter hypermethylation in vivo [17]. Hence we suggest that it may be important to stratify C9-BAC mice based on their epigenetic status while conducting phenotypic assessments and performing pre-clinical testing of experimental therapeutics.

Despite increased levels of repressive histone and DNA methylation marks, expanded C9ORF72 alleles sustain sufficient transcriptional activity to produce toxic levels of mutant RNAs and DPRs. We have recently identified enrichment of 5hmC, an active DNA demethylation intermediate, as a novel epigenetic alteration common to at least two repeat expansion disorders: Fragile X Syndrome and C9-ALS [18, 31]. Given the high abundance of 5hmC at the C9ORF72 promoter of hypermethylated patients and C9-BAC mice, we postulate that active DNA demethylation counteracts the protective hypermethylation of the promoter. Active DNA demethylation is a process facilitated by the ten-eleven translocation (TET) family proteins and acts as a switch between transcriptionally inactive and active gene states [36]. Our data demonstrate that active DNA demethylation is most pronounced in the brain of C9-BAC mice, in concordance with the literature indicating that TET activity and global 5hmC levels are highest in neurons [32, 37]. Future studies are warranted to investigate the possibility of leveraging epigenetic equilibrium, perhaps by using small molecules, in such a way that it favors DNA hypermethylation to more completely repress the expanded locus. This would be hypothesized to reduce the production of toxic products in C9-BAC mice.

The mechanism by which DNA hypermethylation is established at the C9ORF72 promoter has been studied by our group and others [18, 29, 38]. These studies suggest that RNA-DNA hybrids or R-loops are key mediators of epigenetic repression [39]. Recent discoveries in other repeat expansion disorders, including Fragile X Syndrome and Freidriech’s Ataxia support this reasoning [21, 34]. Here we investigated this possibility using iPSC-derived motor neurons, a method that we have successfully utilized in our previous report to model the acquisition of DNA hypermethylation in C9-ALS [18]. We show that despite their presence at the C9ORF72 promoter of C9-ALS patient derived cells, reducing R-loop formation by depleting HRE RNAs is not sufficient to prevent the acquisition of DNA hypermethylation. Future studies could investigate alternative mechanisms leading to the epigenetic repression of HRE promoters. Though we did not address this issue here, it would also be interesting to see whether C9ORF72 knockdown in C9-BAC mice will have any effect on the levels of DNA methylation associated with the expanded locus.

## Conclusions

We have determined that epigenetic perturbations seen in C9-ALS patients are also observed in C9-BAC mice and that R-loops are unlikely to be initiators of DNA methylation at the expanded C9ORF72 locus. Since many repeat expansion mutations, including C9-ALS, have an epigenetic component that is directly linked to pathology, our observations are of significant importance for understanding the mechanism of disease and are of consequence for therapeutic development efforts utilizing C9-BAC mouse models.

## Additional files

Additional file 1: Figure S1. Relative quantification (RQ) values of mouse beta-actin, GAPDH and 18S endogenous controls in C9-BAC mouse cortex across different age groups are shown (A). Absolute copy number of human C9ORF72 transcripts per microliter in C9-BAC mice as determined by digital droplet PCR (B), one-way ANOVA (*p* < 0.001) and Bonferroni’s multiple comparison test was performed between neonatal (0wks) and the remaining age groups, significance is indicated by *p* < 0.05 * and *p* < 0.01 **. (JPEG 238 kb)

Additional file 2: Figure S2. Copy number variation analysis for human C9ORF72 transgene in C9-BAC mouse brain cortex with hypermethylated (me+), unmethylated (me-) promoter and wild-type mouse (WT). (JPEG 142 kb)

Additional file 3: Figure S3. Linear regression analysis of the mean C9ORF72 promoter methylation percentile (as determined by bisulfite pyrosequencing) and glycine-proline dipeptide abundance (A). R square and *p* values for individual CpG dinucleotides are indicated (B). Quantitative PCR assessment of C9ORF72 expression in hypermethylated C9-BAC mice indicated by open shapes (17wks square, 30wks triangle, 36wks circle) and their age group counterparts (C), error bars represent SEM. (JPEG 530 kb)

## Abbreviations

5hmC: 5-hydroxymethylcytosine
5mC: 5-methylcytosine
BAC: Bacterial artificial chromosome
C9-ALS: C9ORF72-associated amyotrophic lateral sclerosis
DPR: Dipeptide-repeat proteins
FTD: Frontotemporal dementia
FXS: Fragile X syndrome
HRE: Hexanucleotide repeat expansion
iPSC: Induced pluripotent stem cells
TSS: Transcription start site
