# Supplementary figures and images for: A C9ORF72 BAC mouse model recapitulates key epigenetic perturbations of ALS/FTD
Source: Mol Neurodegener. 2017 Jun 12;12:46. doi: 10.1186/s13024-017-0185-9 (PMC5468954; doi:10.1186/s13024-017-0185-9)

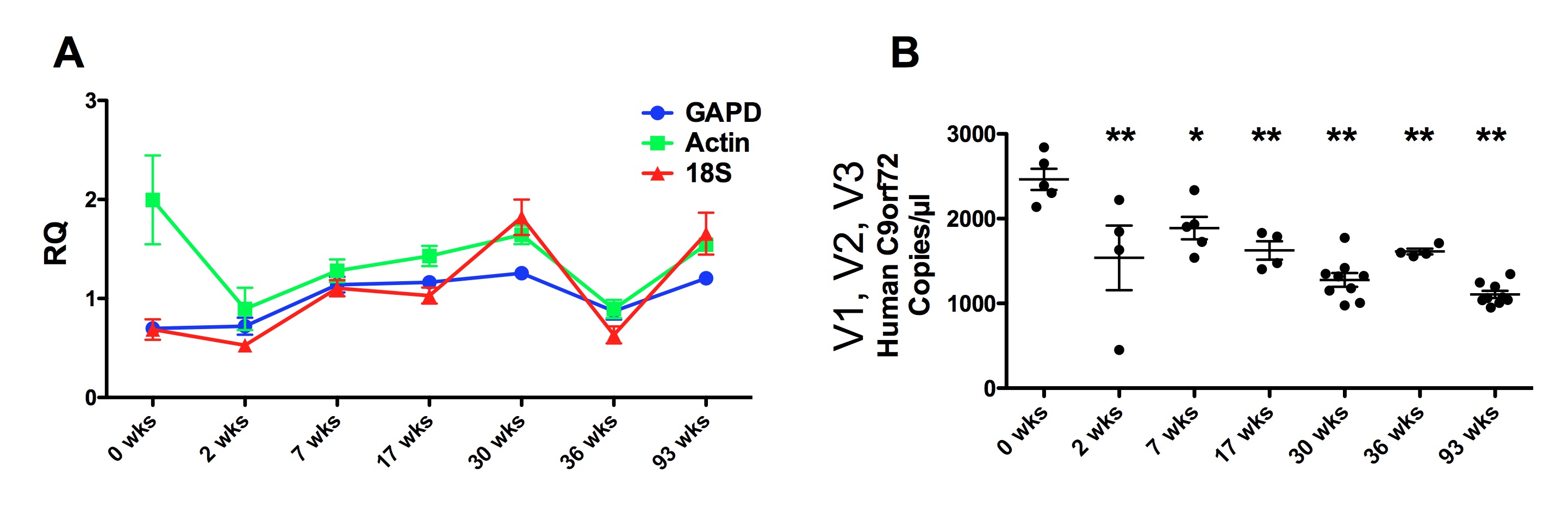

Supplement: Supplementary file 1 — Relative quantification (RQ) values of mouse beta-actin, GAPDH and 18S endogenous controls in C9-BAC mouse cortex across different age groups are shown (A). Absolute copy number of human C9ORF72 transcripts per microliter in C9-BAC mice as determined by digital droplet PCR (B), one-way ANOVA (p < 0.001) and Bonferroni’s multiple comparison test was performed between neonatal (0wks) and the remaining age groups, significance is indicated by p < 0.05 * and p < 0.01 **. (JPEG 238 kb) [file 13024_2017_185_MOESM1_ESM.jpg]

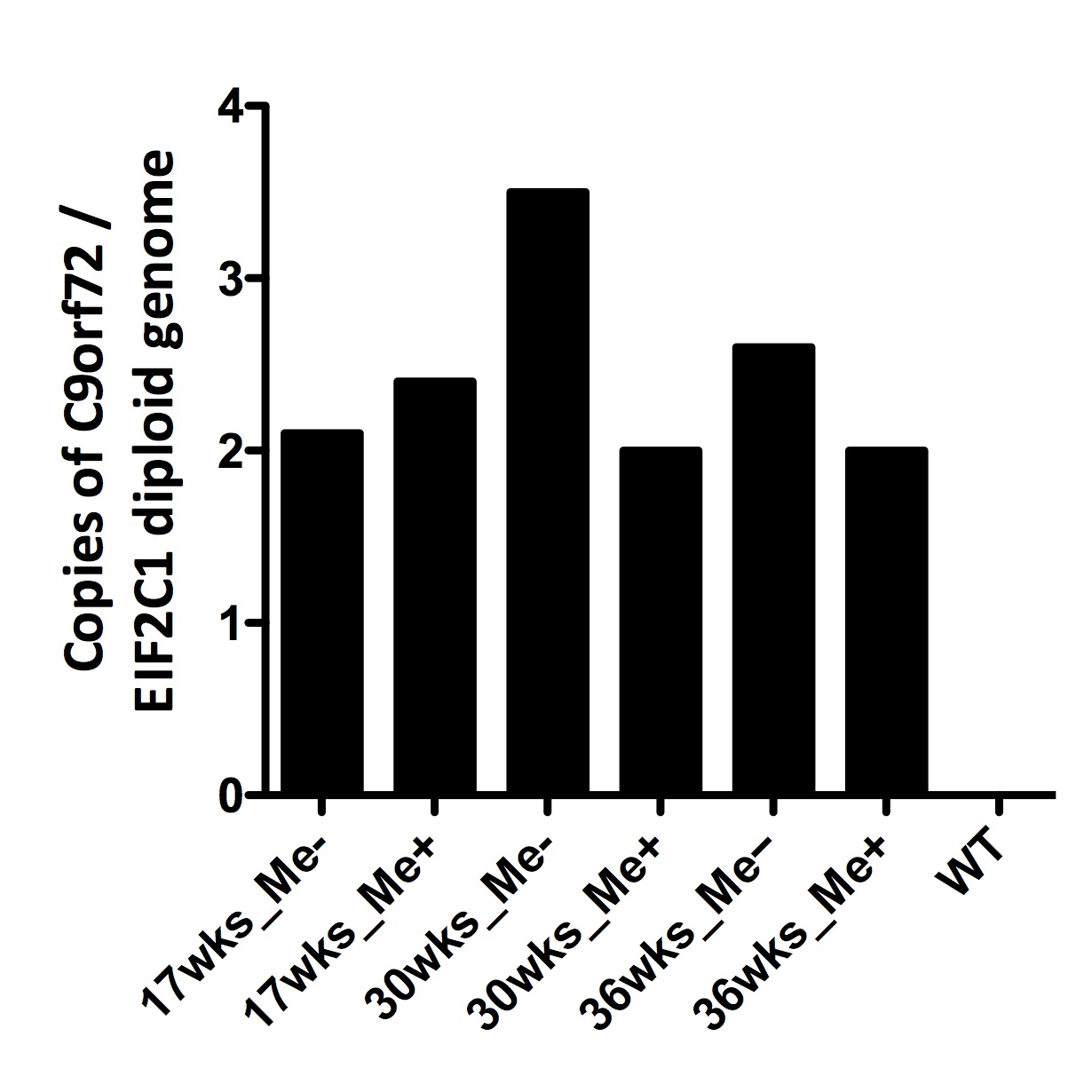

Supplement: Supplementary file 2 — Copy number variation analysis for human C9ORF72 transgene in C9-BAC mouse brain cortex with hypermethylated (me+), unmethylated (me-) promoter and wild-type mouse (WT). (JPEG 142 kb) [file 13024_2017_185_MOESM2_ESM.jpg]

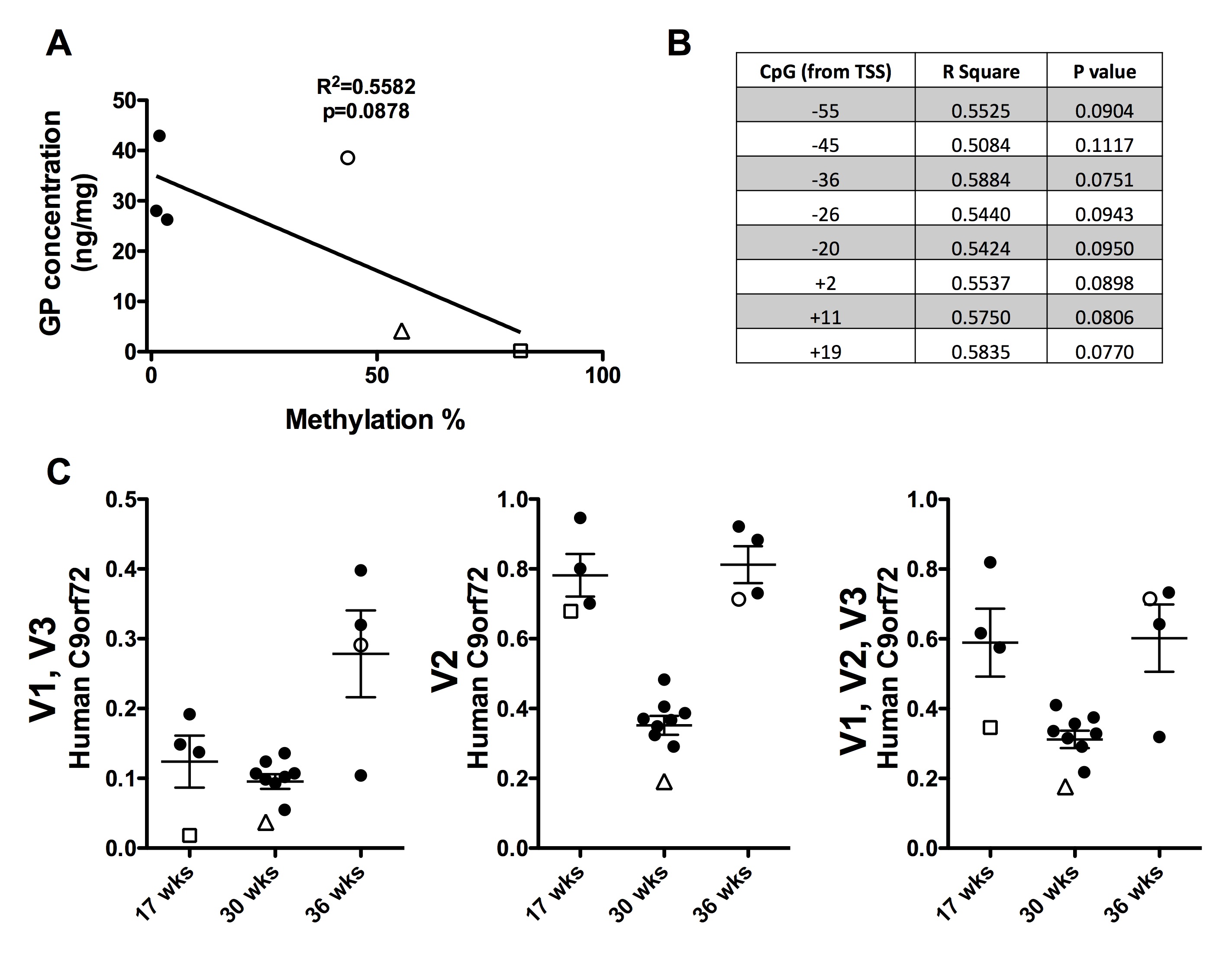

Supplement: Supplementary file 3 — Linear regression analysis of the mean C9ORF72 promoter methylation percentile (as determined by bisulfite pyrosequencing) and glycine-proline dipeptide abundance (A). R square and p values for individual CpG dinucleotides are indicated (B). Quantitative PCR assessment of C9ORF72 expression in hypermethylated C9-BAC mice indicated by open shapes (17wks square, 30wks triangle, 36wks circle) and their age group counterparts (C), error bars represent SEM. (JPEG 530 kb) [file 13024_2017_185_MOESM3_ESM.jpg]
